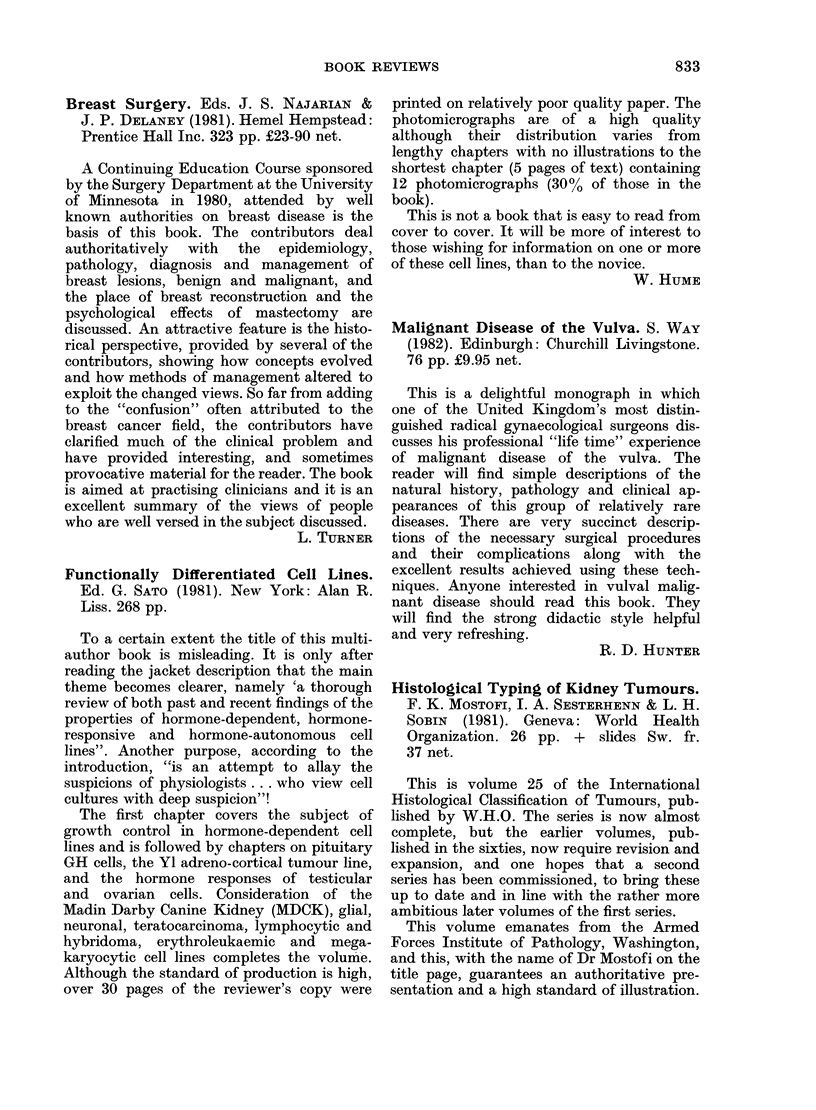# Functionally Differentiated Cell Lines

**Published:** 1982-11

**Authors:** W. Hume


**Functionally Differentiated Cell Lines.** Ed. G. SATO (1981). New York: Alan R. Liss. 268 pp.

To a certain extent the title of this multi-author book is misleading. It is only after reading the jacket description that the main theme becomes clearer, namely 'a thorough review of both past and recent findings of the properties of hormone-dependent, hormone-responsive and hormone-autonomous cell lines". Another purpose, according to the introduction, "is an attempt to allay the suspicions of physiologists . . . who view cell cultures with deep suspicion"!

The first chapter covers the subject of growth control in hormone-dependent cell lines and is followed by chapters on pituitary GH cells, the Y1 adreno-cortical tumour line, and the hormone responses of testicular and ovarian cells. Consideration of the Madin Darby Canine Kidney (MDCK), glial, neuronal, teratocarcinoma, lymphocytic and hybridoma, erythroleukaemic and megakaryocytic cell lines completes the volume. Although the standard of production is high, over 30 pages of the reviewer's copy were printed on relatively poor quality paper. The photomicrographs are of a high quality although their distribution varies from lengthy chapters with no illustrations to the shortest chapter (5 pages of text) containing 12 photomicrographs (30% of those in the book).

This is not a book that is easy to read from cover to cover. It will be more of interest to those wishing for information on one or more of these cell lines, than to the novice.

W. HUME